# Effects of early essential newborn care versus routine birth care on physiological variables and sleep state among newborn infants: a quasi-experimental design

**DOI:** 10.1186/s12887-022-03194-5

**Published:** 2022-03-11

**Authors:** Chuanya Huang, Lei Hu, Jingjing He, Biru Luo

**Affiliations:** 1 West China School of Nursing, Sichuan University / Key Laboratory of Birth Defects and Related Diseases of Women and Children (Sichuan University), Ministry of Education, West China Second University Hospital, Sichuan University, #No. 20, Section 3, People’s South Road, Wuhou District, Chengdu City, 610041 P.R. China; 2 Key Laboratory of Birth Defects and Related Diseases of Women and Children (Sichuan University), Ministry of Education / Department of Nursing, West China Second University Hospital, Sichuan University, #No. 20, Section 3, People’s South Road, Wuhou District, Chengdu, 610000 China

**Keywords:** Early essential newborn care, Physiological stability, Sleep, Infant, newborn, Birth

## Abstract

**Background:**

Early essential newborn care (EENC) was introduced to medical practice in China in 2016, but the number of medical institutions that have put EENC into practice remains low due to insufficient clinical evidence and the absence of awareness among health professionals. This study aimed to explore the effect of EENC on physiological variables and sleep state among newborn infants and to provide evidence to support the implementation of EENC.

**Methods:**

A quasi-experimental design was conducted among 182 newborn infants in a tertiary maternity hospital in China from May 2020 to January 2021. A total of 91 newborn infants were included in the intervention group, and 91 were included in the control group to receive EENC or routine birth care, respectively.

**Results:**

The newborn infants in the intervention group had a lower incidence of hypothermia than those in the control group at 75 min, 90 min, 105 min, and 120 min after birth (*p* < 0.05). The time of first breathing after birth in the intervention group was earlier than that in the control group (5 s vs. 7 s, *p* < 0.05), and the infants had a better sleep state at 30 min, 60 min, 90 min, and 120 min after birth (*p* < 0.05).

**Conclusions:**

EENC can decrease the incidence of hypothermia, promote the initiation of breathing, and improve the sleep state among newborn infants compared to routine birth care in China. More coaching should be provided to health professionals to promote the implementation of EENC in China.

**Trial registration:**

Chinese Clinical Trial Registry, Retrospective Registration (27/7/2021), registration number: ChiCTR2100049231.

## Background

Reducing the mortality rate of newborn infants is a major challenge worldwide, especially in the Western Pacific Region, where a newborn infant dies every 2 minutes [1]. There has been a significant improvement in the survival of children around the world over the last decades; specifically, the number of deaths of under-five children per year dropped from 12.6 million in 1990 to 5.2 million in 2019, while newborn infants accounted for 47% of deaths among under-five children in 2019, up from 40% in 1990 [2]. Approximately one-third of newborn infant deaths occur in the first 24 h after birth [3] and are caused mainly by preterm birth, hypothermia, asphyxia, dyspnoea, and infection [1]. It was estimated that basic neonatal resuscitation and care in the early postnatal periods can reduce the full-term neonatal mortality rate in low-income and middle-income countries by 30% [4]. Therefore, it is of great significance to pay more attention to the provision of early essential newborn care.

The *Action plan for healthy newborn infants in the Western Pacific Region (2014–2020)* was formulated by the World Health Organization Western Pacific Regional Office (WHO/WPRO) in 2013 [1]. The core mission of this action plan is to improve the quality of newborn care services by comprehensively promoting early essential newborn care (EENC). EENC contains a package of evidence-based interventions, including simple and low-cost interventions for all infants during and immediately after birth, such as immediate and thorough drying, immediate and prolonged skin-to-skin contact between mothers and infants, and early exclusive breastfeeding [1, 5]. The WHO pointed out that the implementation of high-quality EENC could effectively prevent prematurity, low birthweight, birth asphyxia, infection, sepsis, and other major causes of newborn morbidity and mortality [6].

Routine birth care has many outdated newborn care practices, such as unnecessary suctioning and immediate cord cutting, while drying and first breastfeeding are delayed, thereby exposing newborn infants to hypothermia, breathing and circulatory problems, infection and brain haemorrhage [1].Compared to routine birth care, EENC especially emphasizes early skin-to-skin contact between the mother and newborns for at least 90 min and early exclusive breastfeeding [7]. To ensure that skin-to-skin contact can be initiated within the first minute after birth, EENC postpones medical services that might interfere with skin-to-skin contact and breastfeeding. The midwife is required to start drying the newborn infant within 5 s after birth, thoroughly dry the infant within 20 to 30 s and appropriately time the clamping and cutting of the cord. Previous studies have shown that skin-to-skin contact can maintain the stability of body temperature, oxygen saturation and heart rate of newborn infants, especially in middle–low-income settings [8, 9]. Furthermore, the study by Karl Bauerprevious et al. showed that newborn infants had a better sleep state during skin-to-skin contact than in the incubator [10]. Sleep is vital for newborn infants because it can facilitate neural development and maturation and optimize physical growth [11, 12]. In addition, drying newborn infants can stimulate the start of breathing in the infant and is recommended by some neonatal resuscitation guidelines [13]. Hence, it is estimated that at least 50,000 newborn deaths can be prevented every year if EENC is fully implemented in medical institutions in the Western Pacific Region [14].

EENC has been introduced and implemented in over 100 medical institutions in China since 2015 [5]. Yang et al. investigated the implementation of EENC in 4 provinces of China in 2019 and showed that 36.24% of mothers and newborn infants had skin-to-skin contact, among whom only 19.7% had skin-to-skin contact for more than 90 min [15]. Additionally, the breastfeeding rate and the exclusive breastfeeding rate before discharge were 76.5 and 32.1%, respectively [15]. A study by Li et al. showed that the exclusive breastfeeding rate was 83.5%, and the rate of a skin-to-skin contact duration for more than 90 min was 38.4% among eight countries in Asia and the Pacific that implemented EENC [4]. Therefore, the status quo of EENC implementation in China is not ideal and needs further improvement. Factors hindering the implementation of EENC in China include the lack of unified EENC implementation guidelines, insufficient support from hospital leaders, a shortage of neonatal health service resources, and a lack of relevant clinical evidence, which leads to the inadequate awareness and acceptance of EENC among obstetricians and midwives [16]. Nevertheless, few studies have focused on EENC in China, and many of the studies that did address EENC were not carried out in full accordance with WHO recommendations [17]. For instance, the duration of skin-to-skin contact between mothers and newborn infants lasted only for 30 min in some studies [18, 19], instead of 90 min. In addition, few published studies were designed as concurrent controlled trials [18, 20, 21], so it is difficult to identify the true clinical effect of EENC. Therefore, studies exploring and validating the clinical effect of high-quality EENC on newborn infants and women are needed. To the best of our knowledge, no study has explored the effect of EENC on physiological variables and sleep state among healthy and full-term newborns delivered vaginally in China. The objective of the current study was to fill this research gap, highlight the necessity, and provide more evidence for the promotion of EENC in the future.

## Methods

### Study design and setting

A quasi-experimental design was implemented with the aim of evaluating the effectiveness of EENC (intervention) compared with routine birth care (control). This study was conducted in a tertiary maternity hospital in Sichuan Province in China from May 2020 to January 2021. This hospital has two labour wards, which were randomly assigned to the intervention group and control group using a table of random numbers. As these two wards are in different hospital areas, any contamination can be excluded. Before the current study, neither ward had implemented EENC or received EENC coaching. In addition, the composition of health professionals was comparable between the two groups, and the facilities of both groups were identical.

### Participants

All pregnant women who were eligible for this study and were willing to provide informed consent were recruited from the maternity ward before birth, and their babies were recruited into the study after birth. In this hospital, the pregnant women chose the labour ward to give birth based on the hospital areas where they received antenatal care. The choice of which hospital area to receive antenatal care depended entirely on the preference of the pregnant woman, such as the consideration for the distance to the hospital area from home. Hence, the participants were nonrandomly assigned into the two groups. However, neither the pregnant women nor their obstetricians knew the group assignment in advance.

The inclusion criteria were as follows: (1) female age ≥ 18 years, (2) gestational age ≥ 37 weeks and < 42 weeks, (3) singleton pregnancy, (4) vaginal delivery, (5) the birth weight of newborn infants was between 2500 g and 4000 g estimated by obstetric ultrasound, and (5) provision of informed consent.

The exclusion criteria were as follows: (1) women with severe pregnancy complications and/or underlying disease, (2) women with medical indications against breastfeeding, such as acquired immunodeficiency syndrome, syphilis, and history of breast surgery, (3) women with no intention to breastfeed, (4) newborn infants with deformities, and (5) newborn infants who needed to be transferred to the neonatal intensive care unit (NICU) immediately after birth. In addition, if the actual birth weight of newborn infants was < 2500 g or > 4000 g, they would be excluded from the data analysis.

The current study is part of a larger trial and focused only on the effect of EENC on the physiological variables and sleep state of newborn infants. The association of EENC with other outcomes will be published separately.

### Intervention

Participants in the intervention group received EENC interventions supported by trained midwives from November 2020 to January 2021. These midwives were trained by national and provincial facilitators from May 2020 to October 2020, following the guidance of *Coaching for the First Embrace: Facilitator’s Guide (Early Essential Newborn Care): Module 2* [22]. Before the actual intervention, a pilot study was carried out on 18 newborn infants (each group comprising 9 newborn infants) to ensure that every midwife in the intervention group had mastered the skills to implement EENC as recommended by the WHO, as well as to obtain data on the incidence of hypothermia among newborn infants. Then, the sample size of this study was calculated. Another objective of the pilot study was to ensure that every data collector could collect and record the data correctly.

The core EENC interventions for all newborn infants are as follows: (1) drying the newborn infant immediately and thoroughly within 5 s after birth, (2) immediate and continuous skin-to-skin contact between the mother and newborn infant for at least 90 min, (3) appropriately timed clamping and cutting of the cord, (4) early exclusive breastfeeding, and (5) routine care, namely, eye care, vitamin K, immunizations, weighing and examinations.

Participants in the control group received routine birth care supported by the midwives. The flow of routine birth care was as follows: (1) drying of the newborn infant, (2) placing of the newborn infant on the radiant warmer to keep warm for 20 min, during which the umbilical cord was clamped and the weight and length were measured, (3) vaccination of the infant, and (4) skin-to-skin contact between the mother and newborn infant and breastfeeding of the infant after the third stage of labour.

The current study was a single-blinded trial. It was impossible to blind the midwives and data collectors in the labour wards. Hence, only participants were blinded.

### Outcomes

This study used a self-designed form to collect the sociodemographic information of participants, including women and their newborn infants. Data on the age, educational level, gestational age, previous obstetric history, height, and weight of the women were collected by data collectors before birth. In addition, data on the sex, length, and birthweight of infants were collected by data collectors after birth.

The primary outcome of the current study was the incidence of hypothermia among newborn infants within the first 2 h after birth. The body temperature of newborn infants was measured with an ear thermometer by the data collectors every 15 min within the first 2 h after birth. If the body temperature of the newborn infant was lower than 36.5 °C, hypothermia was considered [19]. Both body temperature and the incidence of hypothermia were recorded by the data collectors.

The secondary outcomes were the time of first breathing after birth, the incidence of low oxygen saturation, the incidence of abnormal neonatal heart rate, and sleep state in newborn infants. The time of first breathing after birth in the newborn infants was measured with the stopwatch and then recorded by the data collectors. In addition, low oxygen saturation and abnormal heart rates within the first 2 h after birth were measured with the disposable probe cover on pulse oximetry and recorded by data collectors every 10 min. According to the newborn resuscitation guidelines of China [23], the definition of low oxygen saturation in this study was < 80% within 5 min after birth, < 85% within 10 min after birth, and < 90% over 10 min after birth. The definition of abnormal neonatal heart rate was ≤100/min or ≥ 180/min [23]. We used the six sleep and awake states defined by Brazelton (1984), which include deep sleep, light sleep, drowsy, quiet awake, active awake, and agitated fussy, to observe the sleep state of newborn infants [24]. The data collectors were responsible for evaluating the sleep state of newborn infants every 30 min within the first 2 h after birth, with each observation lasting for at least 1 minute.

All data collectors in this study were women with at least a medical-related undergraduate degree. They obtained permission to enter the labour ward and collect data from both the ethics committee of the hospital and the participants. The data collectors received training on methods for collecting data by researchers.

### Sample size

PASS 15 software was used to calculate the sample size. The sample size was estimated according to the primary outcome of this study, which is the incidence of hypothermia within 2 h after birth in the intervention group and the control group. The pilot study showed that the incidences of hypothermia within 2 h after birth were 11.1 and 33.3% in the intervention group and control group, respectively, so the total sample size was 164 mother–newborn pairs (*α* = 0.05, *β* = 0.1). Considering the 10% drop-out rate, the minimum sample size was 182 mother–newborn pairs, with 91 pairs in each group. To reduce sampling error [25], all pregnant women who met the inclusion criteria were included in the study during the recruitment phase.

### Statistical methods

SPSS version 25.0 was used to perform the data analysis. Continuous data with a normal distribution are presented as the mean ± standard deviation (SD), and a t test was then used to analyse significant differences between the two groups. Continuous data with a skewed distribution are presented as the median (interquartile range, IQR), and the Mann–Whitney U test was used to analyse differences between these two groups. Pearson’s chi-square or Fisher’s exact test was used to compare categorical variables. Repeated measures analysis of variance was used to compare the trends of changes in body temperature among newborn infants in both groups across the 2-h post-delivery period. A *p*-value less than 0.05 was considered statistically significant.

## Results

### Flow diagram and baseline characteristics of participants

**Fig. 1:**
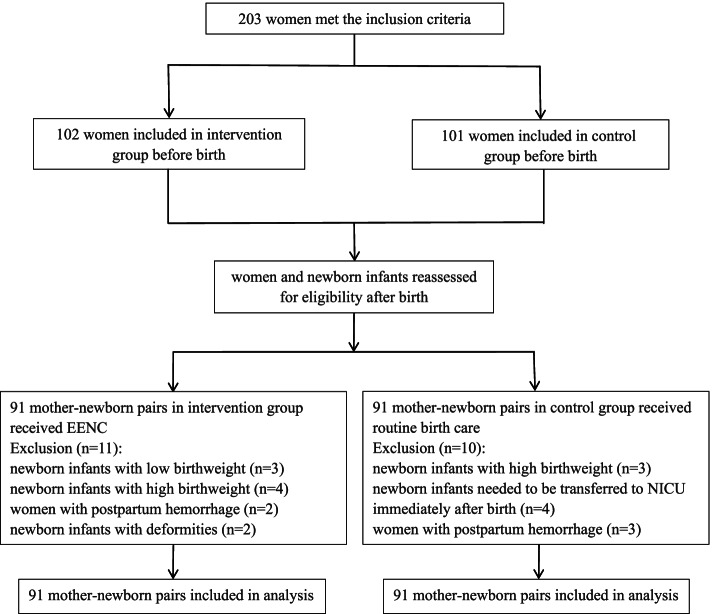
Flow diagram of participants. EENC, early essential newborn care; NICU, neonatal intensive care unit

Between November 2020 and January 2021, 203 pregnant women who met the inclusion criteria were recruited for this study. After exclusions, 182 mother–newborn pairs who received either EENC (91 pairs) or routine birth care (91 pairs) were included at data analysis stage. The flow diagram of participants in this study is shown in Fig. 1. All the demographic characteristics of the mothers and newborn infants were comparable between the two groups (*p* > 0.05), the details of which are listed in Table 1.

**Table 1:** Baseline characteristics of participants

Variables	Intervention group (*n* = 91)	Control group (*n* = 91)	*p* value ^a^
**Maternal**			
Age (years), Mean ± SD	30.23 ± 2.78	30.32 ± 3.27	0.845
Educational level (%)			0.073
High school and below	2 (2.2)	8 (8.8)	
Junior college	14 (15.4)	8 (8.8)	
Undergraduate and above	75 (82.4)	75 (82.4)	
Gestational age (weeks), Mean ± SD	39.63 ± 0.98	39.51 ± 0.97	0.403
Previous obstetric history (%)			0.628
primiparous	65 (71.4)	62 (68.1)	
multiparous	26 (28.6)	29 (31.9)	
Height (m^*^), Mean ± SD	1.60 ± 0.04	1.61 ± 0.05	0.107
Weight (kg^*^), Mean ± SD	64.69 ± 7.82	66.50 ± 7.03	0.102
**Newborn**			
Sex (%)			0.767
Male	47 (51.6)	45 (49.5)	
Female	44 (48.4)	46 (50.5)	
Length (cm^*^), Mean ± SD	49.78 ± 1.58	50.04 ± 1.48	0.245
Birthweight (g^*^), Mean ± SD	3251.65 ± 362.22	3232.53 ± 317.33	0.705

^a^ the two-tailed t test and chi-square test were used for comparisons between the intervention group and the control group

^*^ m, metre; kg, kilogram; cm, centimetre; g, gram

### The incidence of hypothermia and body temperature of newborn infants

The incidence of hypothermia in newborn infants in the intervention group was lower than that in the control group at 75 min (7.7% vs. 17.6%, *p* = 0.045), 90 min (6.6% vs. 22%, *p* = 0.003), 105 min (9.9% vs. 24.2%, *p* = 0.010), and 120 min (8.8% vs. 24.2%, *p* = 0.005) after birth, while it was higher at 15 min (23.1% vs. 9.9%, *p* = 0.017) after birth. There was no significant difference in the incidence of hypothermia of newborn infants at 30 min, 45 min, or 60 min after birth between the two groups (*p* > 0.05) (Table 2).

**Table 2:** The incidence of hypothermia and body temperature at different time points after birth

Time point	Incidence of hypothermia (%)			Body temperature (°C), Mean ± SD					
	Intervention group (*n* = 91)	Control group (*n* = 91)	*p* value ^a^	Intervention group (*n* = 91)	Control group (*n* = 91)	*p* value ^b^	Time, *p* value ^c^	Group, *p* value ^c^	Time × Group, *p* value ^c^
0 min	22 (24.2)	24 (26.4)	0.733	36.64 ± 0.38	36.65 ± 0.35	0.871	< 0.001	0.597	<0.001
15 min	21 (23.1)	9 (9.9)	0.017	36.63 ± 0.28	36.92 ± 0.37	< 0.001			
30 min	18 (19.8)	14 (15.4)	0.436	36.65 ± 0.30	36.79 ± 0.34	0.004			
45 min	15 (16.5)	16 (17.6)	0.844	36.69 ± 0.31	36.77 ± 0.36	0.086			
60 min	10 (11.0)	15 (16.5)	0.282	36.76 ± 0.28	36.75 ± 0.32	0.811			
75 min	7 (7.7)	16 (17.6)	0.045	36.83 ± 0.28	36.73 ± 0.31	0.030			
90 min	6 (6.6)	20 (22.0)	0.003	36.89 ± 0.31	36.71 ± 0.34	< 0.001			
105 min	9 (9.9)	22 (24.2)	0.010	36.89 ± 0.32	36.66 ± 0.32	< 0.001			
120 min	8 (8.8)	22 (24.2)	0.005	36.83 ± 0.31	36.64 ± 0.30	< 0.001			

^a^ chi-square test was used to analyse differences between the intervention group and the control group

^b^ t test was used to analyse differences between the intervention group and the control group

^c^ resulting from repeated measures analysis of variance

**Fig. 2:**
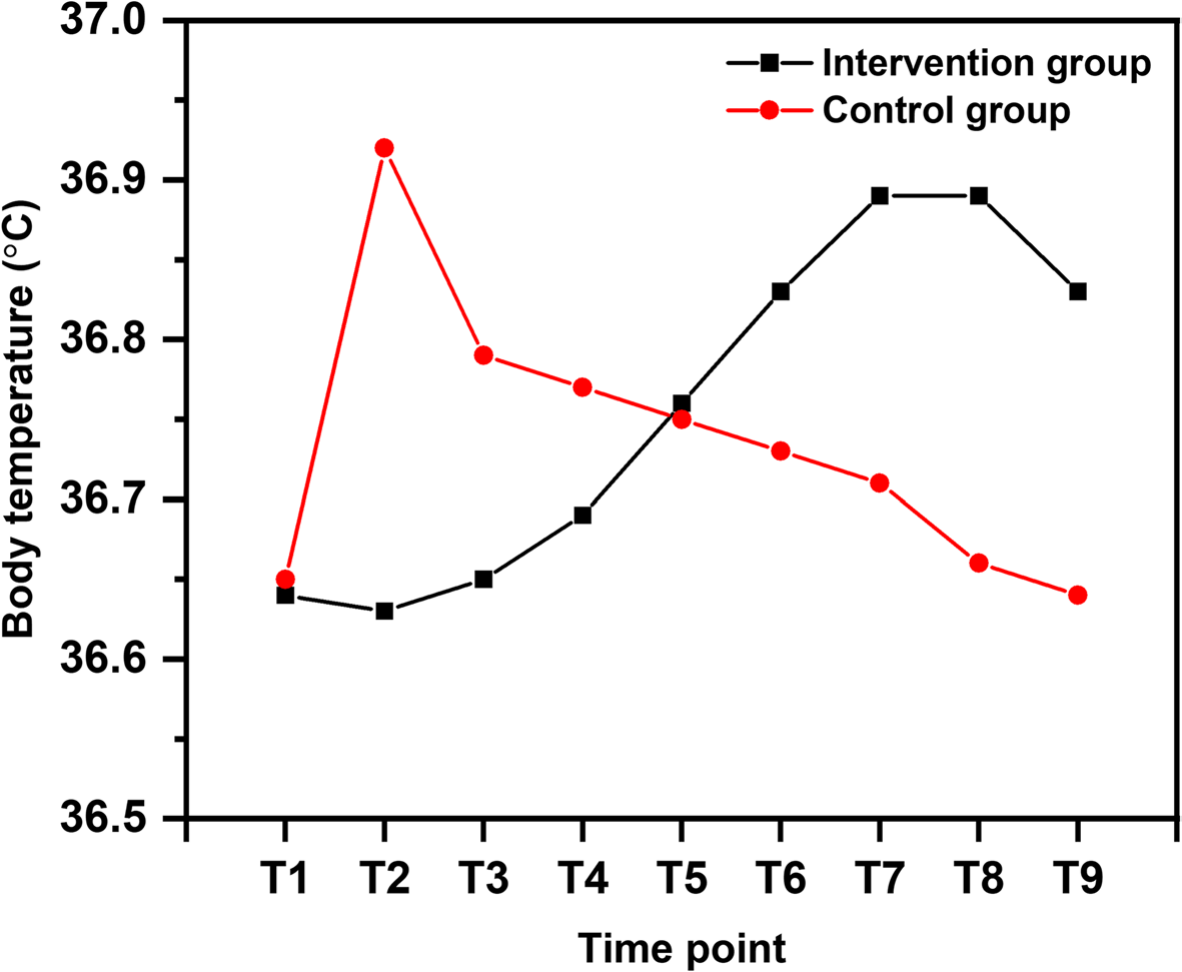
Trend for changes in body temperature of newborn infants in the two groups (T1 = 0 min, T2 = 15 min, T3 = 30 min, T4 = 45 min, T5 = 60 min, T6 = 75 min, T7 = 90 min, T8 = 105 min, T9 = 120 min)

In terms of body temperature (Table 2), there was no significant difference between the two groups at birth or 45 or 60 min after birth (*p* > 0.05). The infant body temperature in the intervention group was lower than that in the control group at 15 min (36.63 ± 0.28 vs. 36.92 ± 0.37, *p* < 0.001) and 30 min (36.65 ± 0.30 vs. 36.79 ± 0.34, *p* = 0.004) after birth but higher than that of the control group at 75 min (36.83 ± 0.28 vs. 36.73 ± 0.31, *p* = 0.030), 90 min (36.89 ± 0.31 vs. 36.71 ± 0.34, *p* < 0.001), 105 min (36.89 ± 0.32 vs. 36.66 ± 0.32, *p* < 0.001), and 120 min (36.83 ± 0.31 vs. 36.64 ± 0.30, *p* < 0.001) after birth. Moreover, the newborn infant body temperature was different at the different time points (*p* < 0.001), and the interaction effect was significant (*p* = 0.034), indicating that newborn infant body temperature was influenced by time and the intervention. The trend for changes in body temperature over time was also different between the two groups (Fig. 2).

### Breathing and heart rates of newborn infants

The newborn infants in the intervention group began to breathe after birth earlier than those in the control group (5 s vs. 7 s, *p* = 0.001). There were 10 (11.0%) and 12 (13.2%) newborn infants who had abnormal heart rates in the intervention group and control group, respectively, within 30 min after birth. Additionally, 5 (5.5%) and 8 (8.8%) newborn infants had low oxygen saturation in these two groups, but no significant difference was found (*p* = 0.649; *p* = 0.388) (Table 3).

**Table 3:** Time of first breathing after birth, heart rate, and oxygen saturation within 2 h after birth

Variables	Intervention group (*n* = 91)	Control group (*n* = 91)	*p* value ^a^
Time of first breathing after birth (seconds), median (IQR)	5 (3, 11)	7 (4, 19)	0.001
Abnormal heart rate, n (%)	10 (11.0)	12 (13.2)	0.649
Low oxygen saturation, n (%)	5 (5.5)	8 (8.8)	0.388

^a^ chi-square test or Mann–Whitney U test was used for comparisons between the intervention group and the control group

### Sleep state of newborn infants

Table 4 shows that there was no significant difference in the sleep state of newborn infants between the two groups at birth (*p* = 0.948). However, at 30 min, 60 min, 90 min, and 120 min after birth, more newborn infants in the intervention group had drowsy, light sleep, and deep sleep states than those in the control group, and the difference was statistically significant (*p* < 0.001), which indicates that the newborn infants in the intervention group had better sleep states than those in the control group after birth.

**Table 4:** Sleep states of newborn infants within 120 min after birth for both groups

Time point	Intervention group (*n* = 91) n (%)	Control group (*n* = 91) n (%)	*p* value ^a^
**0 min**			0.948
agitated fussy	74 (81.3)	76 (83.5)	
active awake	9 (9.9)	9 (9.9)	
quiet awake	6 (6.6)	4 (4.4)	
drowsy	2 (2.2)	2 (2.2)	
**30 min**			< 0.001
agitated fussy	3 (3.3)	8 (8.8)	
active awake	25 (27.5)	38 (41.8)	
quiet awake	29 (31.9)	38 (41.8)	
drowsy	31 (34.1)	7 (7.7)	
light sleep	3 (3.3)	0	
**60 min**			< 0.001
agitated fussy	2 (2.2)	6 (6.6)	
active awake	12 (13.2)	23 (25.3)	
quiet awake	25 (27.5)	40 (44.0)	
drowsy	45 (49.5)	19 (20.9)	
light sleep	6 (6.6)	2 (2.2)	
deep sleep	1 (1.1)	1 (1.1)	
**90 min**			< 0.001
agitated fussy	2 (2.2)	4 (4.4)	
active awake	7 (7.7)	23 (25.3)	
quiet awake	22 (24.2)	33 (36.3)	
drowsy	37 (40.7)	24 (26.4)	
light sleep	20 (22.0)	6 (6.6)	
deep sleep	3 (3.3)	1 (1.1)	
**120 min**			< 0.001
agitated fussy	1 (1.1)	4 (4.4)	
active awake	2 (2.2)	12 (13.2)	
quiet awake	11 (12.1)	23 (25.3)	
drowsy	38 (41.8)	33 (36.3)	
light sleep	30 (33.0)	18 (19.8)	
deep sleep	9 (9.9)	1 (1.1)	

^a^ chi-square test or Fisher’s exact test was sed for comparisons between the intervention group and the control group

## Discussion

This study compared the effect of EENC and routine birth care on the physiological variables and sleep states of newborn infants in a tertiary maternity hospital in Sichuan Province, China, and the results showed that EENC can reduce the incidence of hypothermia, promote breathing initiation, and improve the sleep state of newborn infants.

Neonatal hypothermia is associated with the major causes of neonatal deaths, including severe infections, prematurity, and birth asphyxia [26]. EENC showed better efficiency in preventing the incidence of neonatal hypothermia than routine birth care in this study. Studies by Bystrova et al. [27] and Christensson K et al. [28] also showed that compared to the newborn infants placed in the radiant table the newborn infants who had skin-to-skin contact with mothers for over 90 min had a lower incidence of abnormal body temperature. EENC proposed that thoroughly drying the newborn infant immediately and having skin-to-skin contact after birth can not only minimize the heat taken away by the evaporation of liquids such as amniotic fluid on the surface of the skin but also allow continuous transmission of heat from the mother to the newborn infant. In addition, skin-to-skin contact between the mother and newborn infant can improve the secretion of oxytocin, increase the temperature of maternal breasts, and further provide warmth to newborn infant [29]. Although the trend for change in newborn infant body temperature is in line with the results of Yuan et al.*’s* study [30] in the intervention group, it is different in the control group. In Yuan’s work, the trend newborn infant body temperature in the control group decreased at first and then increased, which is contradictory to the current study, in which the body temperature increased first and then decreased. This may be due to differences in routine birth care and the temperatures of the radiant warmer set in different hospitals. However, the mean body temperatures of the newborn infants at 15 min and 20 min after birth were lower in the intervention group than in the control group. The most reasonable explanation is that the newborn infants were placed on the radiant warmer immediately after the umbilical cord was cut, and the radiant warmer could increase the body temperature quicker than the mother. Nevertheless, the body temperature of the newborn infants in the control group dropped significantly after the newborn infants were moved from the radiant warmer, and the incidence of hypothermia also began to show an upwards trend, which indicates that although the radiant warmer could increase the body temperature quickly, it cannot preserve the body temperature once the newborn infant was moved away from it [31].

Previous studies showed that approximately 8 to 12% of newborn infants initiated breathing after being stimulated [32, 33]. If appropriate stimulation was given, the mortality rate among newborn infants could be reduced by 10% [34]. Newborn resuscitation guidelines in many countries recommend drying newborn infants immediately after birth to stimulate breathing [13, 35, 36]. The current study also adopted the method of drying the newborn infants to stimulate the newborn infants within 5 s after birth to initiate breathing in the intervention group, and the duration of drying was strictly controlled, which is in line with the recommendation of these guidelines. Therefore, the initiation time of breathing in the intervention group was earlier than that in the control group. However, the acceptance of EENC by midwives is one of the difficulties in the implementation of EENC in China [37]. A previous study showed that some hospital staff in China were concerned that skin-to-skin contact between mothers and newborn infants may increase the risk of asphyxia [17]. Based on the findings of this study, there was no difference in the incidence of low oxygen saturation or abnormal heart rate among newborn infants in the intervention group versus the control group, which indicates that the implementation of EENC in clinical practice can be done safely with prior training of personnel for newborn infants.

The levels of corticosteroids and catecholamines in newborn infants increase after birth and lead to stress responses, which could increase the consumption of energy [38]. Sleeping and waking states of newborn infants can reflect the ability to respond to the stimulation and the underlying function of the brain [39]. Through skin-to-skin contact between mothers and newborn infants, EENC can reduce the stimulation of the external cold environment to the newborn infant, activate the sensory nerves of the newborn infants, reduce the excitability of the sympathetic nervous system, achieve the effect of sedation and relaxation [40], and make the newborn enter the sleep state faster. In addition, the touch, voice, smell, and heartbeat of the mother can pacify the newborn infant and make them feel secure [41]. More newborn infants had the drowsy, light sleep, and deep sleep states at 30 min, 60 min, 90 min, and 120 min after birth in the intervention group than in the control group, so the implementation of EENC is conducive to neonatal recovery from the stimulation and maintains a more stable sleep state.

To our knowledge, the current study is the first study to be designed as a controlled study to explore the effect of EENC on physiological variables and the sleep states of newborn infants in China, but there were some limitations that need to be addressed in future work. First, the follow-up period was relatively short, so the long-term effect of the implementation of EENC on newborn infants remains unknown. Second, although the sample size of this study was calculated according to pretrial estimates, the sample size was not large. Third, only one hospital was selected to carry out the study, and the results may not be representative. Last, the results of nonrandomized studies are more likely to be affected by confounding bias, although many attempts were made to avoid them. Therefore, a long-term, multi-centre, and large-sample randomized controlled trial should be considered in the future to explore the clinical effect of EENC and better ensure the health of newborn infants.

## Conclusion

Compared to routine birth care, EENC can better maintain the body temperature of the newborn infant within the normal range, reduce the incidence of hypothermia, promote the establishment of breathing, and make their sleep state more stable within 2 h after birth. To provide a healthy start for every newborn infant and improve newborn health, implementing high-quality EENC is recommended to policy makers, hospital managers and medical professionals in China.

## Data Availability

All raw data generated or analysed during this study are available from the corresponding author upon reasonable request.

## Abbreviations

EENC: Early Essential Newborn Care
IQR: Inter Quartile Range
WHO: World Health Organization
SD: Standard deviation
NICU: Neonatal intensive care unit
WPRO: Western Pacific Regional Office
